# Spatial patterns and climate drivers of malaria in three border areas of Brazil, Venezuela and Guyana, 2016–2018

**DOI:** 10.1038/s41598-022-14012-4

**Published:** 2022-06-29

**Authors:** Kinley Wangdi, Erica Wetzler, Horace Cox, Paola Marchesini, Leopoldo Villegas, Sara Canavati

**Affiliations:** 1 Department of Global Health, National Centre for Epidemiology and Population Health, ANU College of Health and Medicine, The Australian National University, Canberra, ACT 2601 Australia; 2 World Vision US, 34834 Weyerhaeuser Way South, Federal Way, Washington, USA; 3 Vector Borne Diseases Unit, Guyana Ministry of Health, Georgetown, Guyana; 4 Department of Surveillance for Zoonotic and Vector Borne Diseases, Malaria Technical Group, Ministry of Health, Brasilia, Federal District, Brazil; 5 Global Development One, Silver Spring, MD USA; 6 Asociación Civil Impacto Social (ASOCIS), Tumeremo, Bolívar, Venezuela

**Keywords:** Diseases, Risk factors

## Abstract

In 2020, 77% of malaria cases in the Americas were concentrated in Venezuela, Brazil, and Colombia. These countries are characterized by a heterogeneous malaria landscape and malaria hotspots. Furthermore, the political unrest in Venezuela has led to significant cross-border population movement. Hence, the aim of this study was to describe spatial patterns and identify significant climatic drivers of malaria transmission along the Venezuela-Brazil-Guyana border, focusing on Bolivar state, Venezuela and Roraima state, Brazil. Malaria case data, stratified by species from 2016 to 2018, were obtained from the Brazilian Malaria Epidemiology Surveillance Information System, the Guyana Vector Borne Diseases Program, the Venezuelan Ministry of Health, and civil society organizations. Spatial autocorrelation in malaria incidence was explored using Getis-Ord (Gi*) statistics. A Poisson regression model was developed with a conditional autoregressive prior structure and posterior parameters were estimated using the Bayesian Markov chain Monte Carlo simulation with Gibbs sampling. There were 685,498 malaria cases during the study period. *Plasmodium vivax* was the predominant species (71.7%, 490,861). Malaria hotspots were located in eight municipalities along the Venezuela and Guyana international borders with Brazil. *Plasmodium falciparum* increased by 2.6% (95% credible interval [CrI] 2.1%, 2.8%) for one meter increase in altitude, decreased by 1.6% (95% CrI 1.5%, 2.3%) and 0.9% (95% CrI 0.7%, 2.4%) per 1 cm increase in 6-month lagged precipitation and each 1 °C increase of minimum temperature without lag. Each 1 °C increase of 1-month lagged maximum temperature increased *P. falciparum* by 0.6% (95% CrI 0.4%, 1.9%). *P. vivax* cases increased by 1.5% (95% CrI 1.3%, 1.6%) for one meter increase in altitude and decreased by  1.1% (95% CrI 1.0%, 1.2%) and 7.3% (95% CrI 6.7%, 9.7%) for each 1 cm increase of precipitation lagged at 6-months and 1 °C increase in minimum temperature lagged at 6-months. Each 1°C increase of two-month lagged maximum temperature increased *P. vivax* by 1.5% (95% CrI 0.6%, 7.1%). There was no significant residual spatial clustering after accounting for climatic covariates. Malaria hotspots were located along the Venezuela and Guyana international border with Roraima state, Brazil. In addition to population movement, climatic variables were important drivers of malaria transmission in these areas.

## Introduction

The WHO World Malaria Report 2021 showed that there were an estimated 241 million malaria cases and 627,000 malaria deaths worldwide in 2020. This represents about 14 million more cases in 2020 compared to 2019, and 69,000 more deaths. Approximately two-thirds of these additional deaths (47,000) were linked to disruptions in the provision of malaria prevention, diagnosis and treatment during the pandemic^1^.

In the WHO Region of the Americas, malaria cases and case incidence reduced by 58% (from 1.5 million to 0.65 million) and 67% (from 14.1 to 4.6 cases per 1000 population at risk) between 2000 and 2020^1^. Over the same period, there was reduction in both malaria deaths and the malaria mortality rate by 56% (from 909 to 409) and 66% (from 0.8 to 0.3 deaths per 100,000 population at risk), respectively^1^. However, progress in this region suffered in recent years because of a major increase in malaria in the Bolivarian Republic of Venezuela, which had about 35,500 cases in 2000, rising to over 467,000 by 2019^1^. In 2020, cases reduced to 232,000, or about  half of 2019 cases^1^. This was attributed to restrictions on movement during the COVID-19 pandemic and fuel shortages leading to reduced mining activities. As a result, occupational exposure risk to malaria vectors was significantly decreased^1^.

So far, in the region, Argentina, Paraguay and El Salvador have eliminated malaria^1–3^. Belize reported zero malaria cases for the second consecutive year in 2020^1^. In addition, French Guiana, Guatemala, Honduras and Peru all met the global technical strategy 2020 malaria morbidity milestone of a reduction of at least 40% in case incidence^1^. However, this progress has stalled in some places in recent years, with the rise in cases mainly due to the major increase in malaria in Venezuela^2,4^. The country has been under a severe economic, political, and social crisis and all national institutions have been affected. The collapse of the Venezuelan health system has resulted in the deterioration of all facets of malaria prevention and control^5,6^. Stock-outs of antimalarial drugs have been common, exacerbating malaria transmission^5^.

Furthermore, the political unrest in Venezuela has led to significant cross-border population movement^7^. More than 5.2 million people have left the country since 2015, and there has been a marked influx of Venezuelan nationals arriving in neighboring countries^7^. Malaria transmission in the WHO Region of the Americas is heterogeneous^8,9^ and four countries accounted for 77% of malaria cases in 20,202^1^. Several factors are responsible for the continued transmission of malaria including climatic, ecological and human factors, further characterized by spatial clustering of cases in transmission hotspots^10,11^. If malaria control interventions in hotspot areas are not sustained, these hotspots can serve as sources of infection to neighbouring regions and to countries that have eliminated malaria or where transmission has been interrupted^12,13^. Delineation of malaria hotspots can help to identify the underlying reasons for higher incidence of malaria in particular areas^14^, which can serve to target interventions where they are most needed, likely having a greater impact than uniform resource allocation^11^.

Spatial analysis and modelling enable the prediction of disease patterns and determination of ecological associations between disease risk and the environment^11,15,16^. It is well known that geospatial methods can be used to link disease data to vector habitats, vector presence, abundance and density; quantify spatial diffusion; and characterize spatial and temporal patterns of disease^17–24^. This paper aimed to describe spatial patterns and climatic drivers of malaria from 2016 to 2018 in Brazil (Roraima state), Venezuela (Bolivar state) and four regions of Guyana, all located in the Guyana Shield.

## Methods

### Study area and data

**Figure 1:**
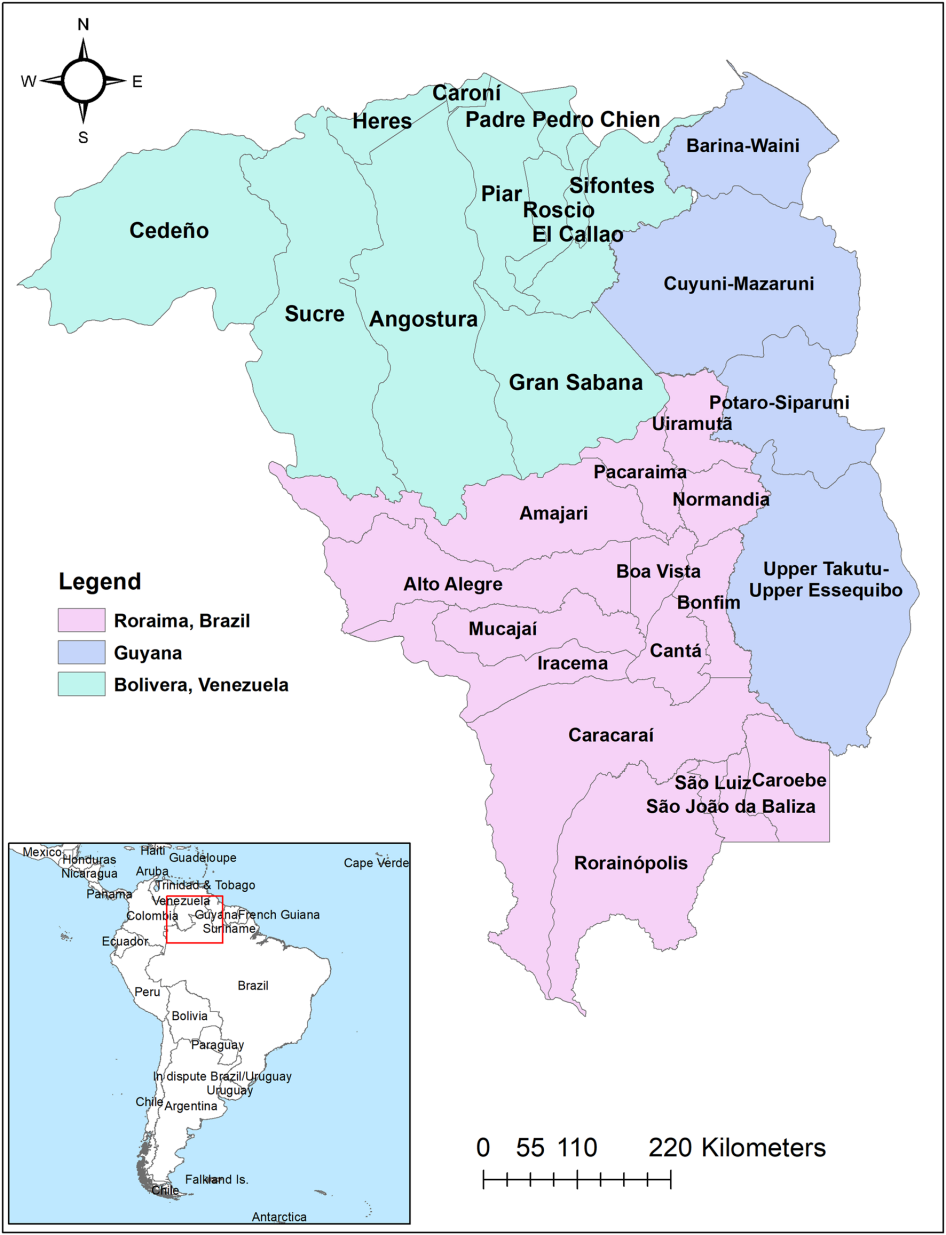
Map of the study areas.

The study area included three border areas: Roraima state in Brazil, Bolivar state in Venezuela and four regions of Guyana (Fig. 1). Roraima and Bolivar are divided into 15 and 11 municipalities, respectively. Individual-level, de-identified datasets were obtained from national surveillance systems and additional sources: the Brazilian Malaria Epidemiology Surveillance Information System (SIVEP-Malaria), the Guyana Vector Borne Diseases Program, the Venezuelan Ministry of Health, and civil society organizations in Venezuela. Individual-level data were extracted on age, sex and malaria species. The populations of the municipalities were obtained from national census projections in each country^25,26^. Monthly precipitation, and minimum and maximum temperature at 2.5 min intervals from January 2016 to December 2018 were obtained from the WorldClim database^27^. Municipality polygon was used to extract the mean climatic variables using Zonal statistics in ArcMap 10.5.1 (ESRI Inc., Redlands, CA, USA). An electronic map of municipalities in shapefile format was obtained from the DIVA-GIS database (https://www.diva-gis.org/).

### Hotspot analysis

The presence and nature of spatial autocorrelation that suggest malaria case clustering by place of notification were assessed by the Getis-Ord statistic (Gi*)^28,29^. The local Getis-Ord statistic (Gi*) was used to identify the intensity and stability of hotspot/cold spot clusters^29,30^. The Gi* statistic compares the local malaria mean rate (i.e., the rate of malaria for a target location and its neighbors) to the global malaria mean rate (the rates of all municipalities). The Gi* statistic compares the z-score and *p*-value for each municipality with global malaria means. Location with a statistically significant and larger z-score will have a more intense clustering of high values (hotspots), where it is unlikely that the spatial clustering of high values is the result of a random spatial process; and locations with statistically significant and smaller z-scores will have more intense clustering of low values (cold spots)^29^. ArcMap 10.5.1 software (ESRI, Redlands, CA) was used for hotspot analysis and creating maps.

### Crude standardized morbidity ratios

An initial descriptive analysis of malaria incidence was conducted. Crude standardized morbidity ratios (SMRs) for each municipality were calculated using the following formula:

$${Y}_{i}= \frac{{O}_{i}}{{E}_{i}}$$

where *Y* is the overall SMR in municipality *i, O* is the total number of observed malaria cases in the municipality and *E* is the expected number of malaria cases in the municipality across the study period. The expected number was calculated by multiplying the national incidence by the average population for each municipality over the study period.

### Independent variable selection

A preliminary Poisson regression was undertaken to select climatic covariates for each species. Climatic variables of precipitation, minimum and maximum temperature without a lag, and with one to 7-month lag times were entered into univariate models. Minimum temperature without lag, 1-month lagged maximum temperature and 6-month lagged precipitation had the lowest values of the Akaike’s information criterion (AIC) for *P. falciparum* (Supplementary Table S1). Two-month maximum temperature and 6-month lagged precipitation and minimum temperature were selected for *P. vivax* with the lowest AIC (Supplementary Table S2). The co-linearity of the climatic variables was tested using variance inflation factors (VIF) (Supplementary Tables S3, S4).

### Spatio-temporal model

Poisson regression models were developed in the Bayesian statistical software WinBUGS version 1.4 (Medical Research Council, Cambridge, UK and Imperial College London, UK) for *P. falciparum* and *P. vivax*. Alternative models were tested for each species including models with climatic variables such as precipitation, minimum and maximum temperature as explanatory variables, and spatially structured and unstructured random effects. The best model was selected based on the lowest deviation information criterion (DIC) for each species. Three models were developed: Model I consisted of climatic explorative variables and unstructured random effects; Model II contained the same explorative variables as Model I and spatially structured random effects. Model III contained both structured and unstructured random effects and climatic explorative variables. Model III was the most comprehensive model, which had as an outcome the observed counts of malaria, *Y*, for *i*^th^ municipality (*i* = 1…30) in the *j*^th^ month (January 2016-December 2018) was structured as follows:

$$Y_{ijkl} \sim {\text{Poisson}}\;{(}\mu_{ijkl} {)}$$

$${\text{log(}}\upmu _{ijkl} {)} = {\text{log(E}}_{ijkl} {)} + \theta_{ijkl}$$

$$\theta_{ijkl} = \alpha + \beta_{1} \times {\text{trend}} + \beta_{2} \times {\text{Altitude}} + \beta _{3} \times {\text{Precipitation}}_{ij} + \beta_{4} \times {\text{Tempmin}}_{ij} + \beta_{5} \times {\text{Tempmax}}_{ij} + {\text{u}}_{i} + {\text{s}}_{i} .$$

where E is the expected number of cases (acting as an offset to control for population size) and θ is the mean log relative risk (RR); α is the intercept, and *β*_1_, *β*_2_, *β*_3_, *β*_4_ and *β*_5_ the coefficients for trend, altitude, precipitation, minimum and maximum temperature, respectively; u_*i*_ is the unstructured random effect (assumed to have a mean of zero and variance σ_u_^2^) and s_*i*_ is the spatially structured random effect (assumed to have a mean of zero and variance σ_s_^2^).

A conditional autoregressive (CAR) prior structure was used to model the spatially structured random effect. An adjacency weights matrix was used to calculate the spatial relationships between the municipalities. A weight of 1 was assigned if two municipalities shared a border and 0 if they did not. A flat prior distribution was specified for the intercept, whereas a normal prior distribution was specified for the coefficients. The priors for the precision of unstructured and spatially structured random effects were specified using non-informative gamma distributions with shape and scale parameters. Models were also developed without the structured and unstructured random effects to assess whether inclusion of these components improved model fit.

An initial burn-in of 10,000 iterations was run, and these iterations were discarded. Subsequent blocks of 20,000 iterations were run and examined for convergence. Convergence was assessed by visual inspection of posterior density and history plots, and occurred at approximately 100,000 iterations for each model. Ten thousand values from the posterior distributions of each model parameter were stored and summarized for the analysis (posterior mean and 95% credible intervals [CrI]).

In all analyses, an α-level of 0.05 was adopted to indicate statistical significance (as indicated by 95% CrI for RR that excluded 1). ArcMap 10.5.1 software (ESRI, Redlands, CA) was used to generate maps of the posterior means of the unstructured from the three models.

### Ethics approval and consent to participate

The National Center of Bioethics in Venezuela (CENABI) approved the research protocol and the National Survey Ethics Council (CONEP) considered that ethical clearance for the use of this secondary data in Brazil was not necessary. Not applicable. Human participants were not involved in the study. This research uses secondary data and is not subject to ethics approval.

## Results

### Descriptive analysis

**Figure 2:**
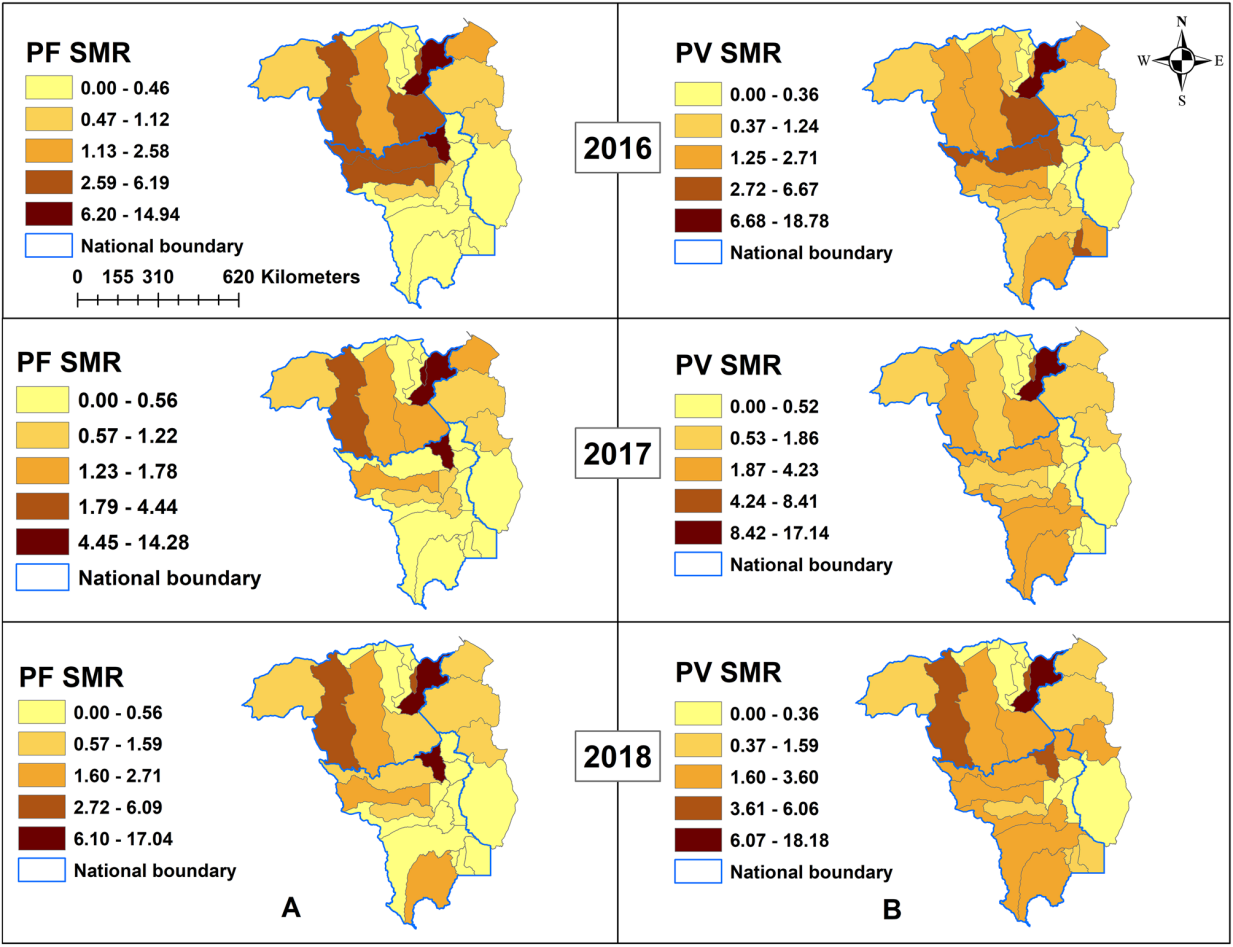
Raw standardized morbidity ratios of (**A**) *Plasmodium falciparum*. (**B**) *Plasmodium vivax*, 2016–2018. PF, *Plasmodium falciparum*; PV, *Plasmodium vivax*; SMR, standardized morbidity ratios.

There were a total of 684,498 malaria cases recorded during the study period, and 88.3% (604,306) of these cases were reported from Bolivar state, Venezuela. Compared to 2016, malaria cases across the study areas increased from 25.5% (174,635) to 37.5% (256,482) and 37.0% (253,381) cases in 2017 and 2018, respectively. More than two-thirds of cases were in males (67.5%, 461,912). The predominant species was *P. vivax* (71.7%, 490,861) followed by *P. falciparum* (22.4%, 153,512)*.* However, mixed infections with *P. vivax* and *P. falciparum* consisted of 4.6% (8,008), 5.7% (14,492) and 6.9% (17,381) in 2016, 2017 and 2018, respectively (Table 1). Mean precipitation, and minimum and maximum temperature were 150.5 mm (range 1.1–413.6 mm), 21.9 °C (range 16.9–24.6 °C) and 31.1 °C (range 26.8–35.2 °C), respectively (Supplementary Table S5). The *P. falciparum* SMR varied from 0.0 to 14.94 during the study period, and Pacaraima municipality in Roraima state and Sifontes municipality in Bolivar state reporting the highest SMR with ranges increasing each year: 2016 (SMR 6.20–14.94), 2017 (SMR 4.45–14.28) and 2018 (SMR 6.1–17.04) (Fig. 2A). The *P. vivax* SMR varied from 0.0 to 18.78 during the study period with Sifontes municipality again reporting the highest SMR: 2016 (SMR 6.68–18.78), 2017 (SMR 8.42–17.14) and 2018 (SMR 6.07–18.18) (Fig. 2B).

**Table 1:** Demographic characteristics of malaria from 2016 to 2018.

Characteristics	Year			*p*-value*
	2016	2017	2018	
**Country**	**Number (%)**	**Number (%)**	**Number (%)**	
Roraima, Brazil	8969 (5.1)	14,082 (5.5)	23,369 (9.2)	< 0.001
Guyana	8355 (4.8)	11,139 (4.3)	14,278 (5.6)	
Bolivar, Venezuela	157,311 (90.1)	23,1261 (90.2)	215,734 (85.1)	
Total	174,635 (25.5)	256, 482 (37.5)	253, 381 (37.0)	
**Sex**				
Female	53,839 (30.8)	83,136 (31.4)	85,611 (33.8)	< 0.001
Male	120,796 (69.2)	173,346 (76.6)	167,770 (66.2)	
**Age category (years)**				
0–18	41,123 (23.7)	62,715 (24.5)	61,618 (24.4)	< 0.001
19–30	63,891 (36.8)	89,782 (56.0)	89,926 (35.6)	
31–40	34,107 (19.6)	50,397 (19.7)	50,311 (19.9)	
40+	34,597 (19.9)	52,797 (20.7)	50,914 (20.1)s	
**Species**				
*P. falciparum*	37,217 (21.3)	53,138 (20.7)	63,157 (24.9)	< 0.001
*P. vivax*	129,354 (74.1)	188,755 (73.6)	172,752 (68.2)	
*P. malariae*	11 (0.0)	13 (0.0)	15 (0.0)	
Mixed	8008 (4.6)	14,492 (5.7)	17,381 (6.9)	

*p-value significant at < 0.05.

**Figure 3:**
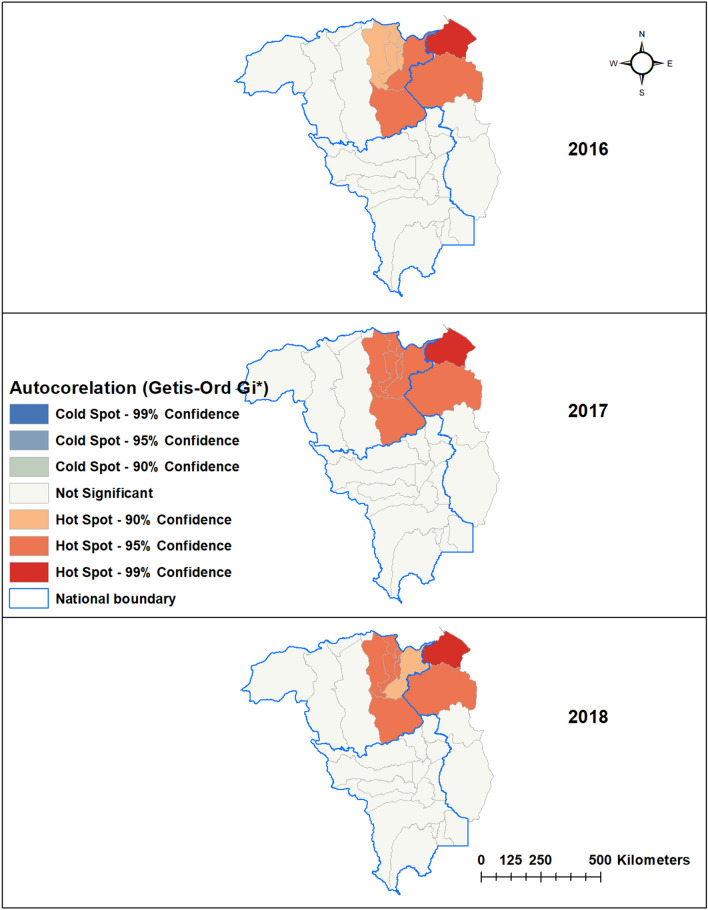
Hot spots (Getis-Ord Gi*) of *Plasmodium falciparum*.

### Spatial autocorrelation

**Figure 4:**
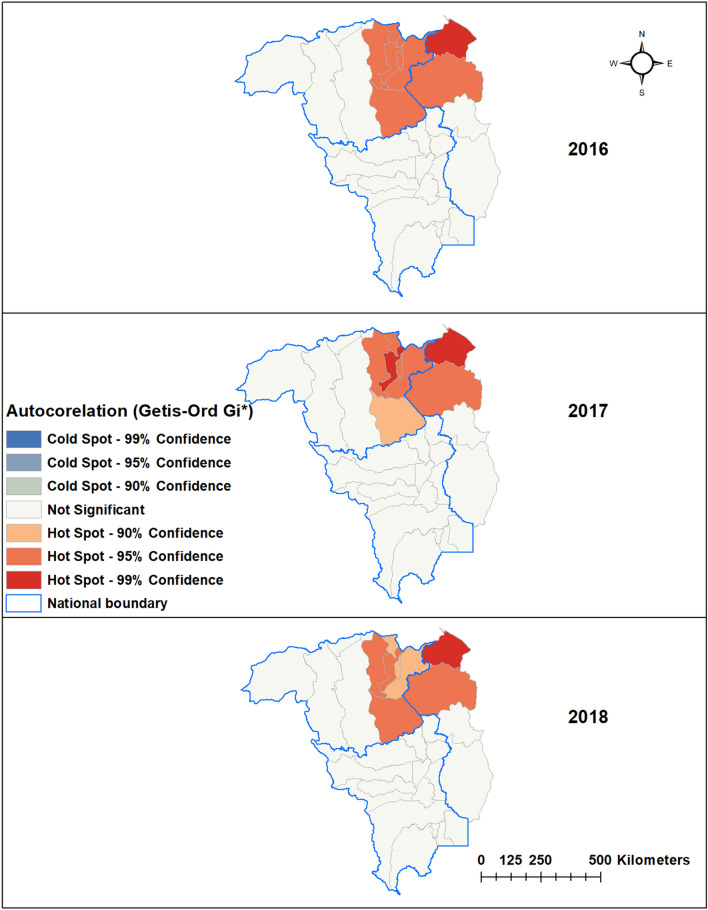
Hot spots (Getis-Ord Gi*) of *Plasmodium vivax.*

Applying the Getis-Ord G* statistic hotspot analysis to *P. falciparum* and *P. vivax* incidence in each municipality revealed statistically significant (*p* < 0.01) hotspots in eight border municipalities of Venezuela and Guyana: Piar, Padre Pedro Chien, Roscio, El Callao and Sifontes in Bolivar state and Barina-Waini and Cuyuni-Mazaruni in Guyana (Figs. 3, 4).

**Figure 5:**
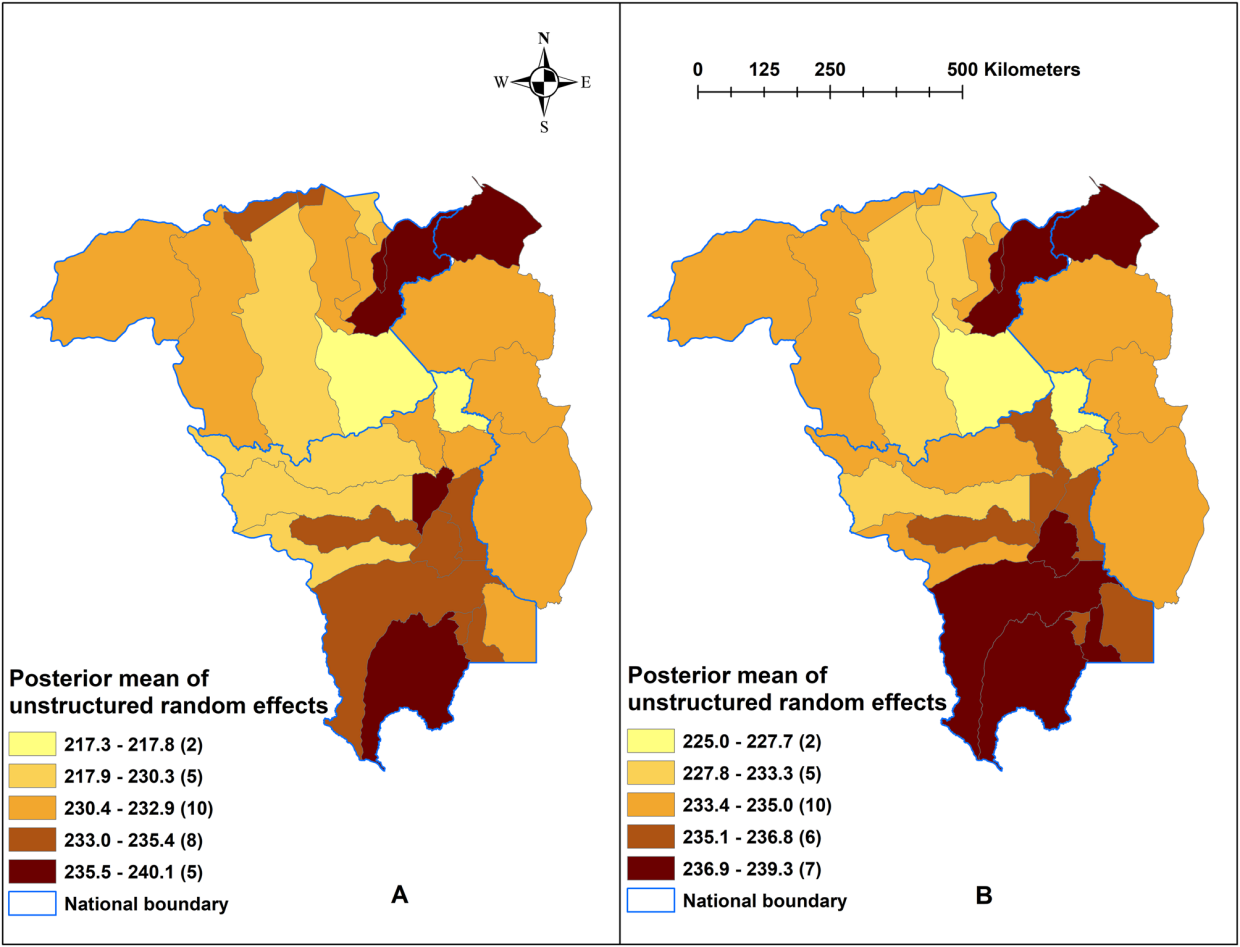
Spatial distribution of the posterior means of unstructured random effects for (**A**) *Plasmodium falciparum* and (**B**) *Plasmodium vivax* in Model I.

### Spatio-temporal model

Model I, containing the unstructured random effect, had the best fit and was the most parsimonious of all the models, examined for both *P. falciparum* and *P. vivax*, as indicated by the lowest DIC (Table 2). For *P. falciparum*, monthly malaria cases increased by 2.1% (95% CrI 2.0%, 2.1%) per month during the study period. One meter increase in altidude increased malaria cases by 2.6% (95% CrI 2.1%, 2.8%). While one cm increase in 6-month lagged precipitation and each 1 °C increase of minimum temperature was associated with 1.6% (95% CrI 1.5%, 2.3%) and 0.9% (95% CrI 0.7%, 2.4%) decrease of *P. falciparum*, respectively. Each 1 °C increase of 1-month lagged maximum temperature increased *P. falciparum* by 0.6% (95% CrI 0.4%, 1.9%). Monthly *P. vivax* increased by 1.0% (95% CrI 0.9%, 1.0%) during the study period. *P. vivax* cases increased by 1.5% (95% CrI 1.3%, 1.6%) for one meter increase in altitude and decreased by 1.1% (95% CrI 1.0%, 1.2%) for each cm increase of precipitation lagged at 6-months and 7.3% (95% CrI 6.7%, 9.7%) for each 1 °C increase in minimum temperature lagged at 6-months. Each 1°C increase of two-month lagged maximum temperature increased *P. vivax* by 1.5% (95% CrI 0.6%, 7.1%) (Table 2). There is no evidence of spatial clustering after accounting for model covariates,- meaning covariates in the model explained the transmission of malaria and there was no effect from malaria transmission in the adjoining municipalities (Fig. 5).

**Table 2:** Regression coefficients, relative risks and 95% CrI from Bayesian spatial and non-spatial models for *Plasmodium falciparum* and *Plasmodium vivax* from 2016 to 2018.

Model/variables	*Plasmodium falciparum* RR (95% CrI)	*Plasmodium vivax* RR (95% CrI)
**Model I**		
Monthly trend	1.021 (1.019, 1.021)	1.010 (1.009, 1.010)
Altitude (m)	1.026 (1.021, 1.028)	1.015 (1.013, 1.016)
Precipitation (10 mm)*	0.984 (0.977, 0.985)	0.989 (0.988, 0.990)
Temp min (°C)*	0.991 (0.976, 0.993)	0.927 (0.903, 0.933)
Temp max (°C)**	1.006 (1.004, 1.019)	1.015 (1.006, 1.071)
Heterogeneity		
Unstructured	1.91 × 10^−5^ (1.08 × 10^−5^, 2.98 × 10^−5^)	1.88 × 10^−5^ (1.06 × 10^−5^, 2.91 × 10^−5^)
DIC^‡^	50,219	124,173
**Model II**		
Monthly trend	1.021 (1.020, 1. 021)	1.010 (1.009, 1.010)
Altitude (m)	1.013 (1.003, 1.021)	1.009 (1.004, 1.014)
Precipitation (10 mm)*	0.984 (0.977, 0.985)	0.989 (0.988, 0.990)
Temp min (°C)**	0.908 (0.776, 0.927)	0.928 (0.901, 0.934)
Temp max (°C)**	1.063 (1.045, 1.216)	1.014 (1.007, 1.077)
Heterogeneity		
Structured (spatial)	3.77 × 10^−2^ (1.08 × 10^−2^, 1.03 × 10^−1^)	6.17 × 10^−2^ (2.10 × 10^−2^, 1.43 × 10^−1^)
DIC	50,888.2	124,413
**Model III**		
Monthly trend	1.021 (1.020, 1.021)	1.016 (1.009, 1.010)
Altitude (m)	1.030 (1.028, 1.032)	1.018 (1.014, 1.021)
Precipitation (10 mm)*	0.984 (0.978, 0.985)	0.989 (0.988, 0.990)
Temp min (°C)**	0.906 (0.774, 0.927)	0.928 (0.905, 0.934)
Temp max (°C)***	1.063 (1.047, 1.217)	1.015 (1.007, 1.074)
Heterogeneity		
Unstructured	2.66 × 10^−4^ (1.49 × 10^−4^, 4.19 × 10^−4^)	3.51 × 10^−4^ (1.99 × 10^−4^, 5.47 × 10^−4^)
Structured (spatial)	9.77 × 10^−4^ (5.28 × 10^−5^, 1.57 × 10^−4^)	1.25 × 10^−4^ (6.93 × 10^−5^, 1.98 × 10^−4^)
DIC	51,059.5	124,422

CrI, credible interval; DIC, deviation information criteria; RR, relative risk.

^‡^Best fit model.

*Precipitation lagged at 6-months for both *Plasmodium falciparum* and *P. vivax.*

**No lag and 6-months lag for *P. falciparum* and *P. vivax.*

***1 and 2-months lagged for *P. falciparum* and *P. vivax.*

## Discussion

This study aimed to describe spatial patterns and climatic drivers of malaria in the border states of Brazil, Venezuela and Guyana using national malaria surveillance data from 2016 to 2018. The great majority of malaria cases were reported from Bolivar state in Venezuela: 157,311 (90.1%), 231,261 (90.2%), and 215,734 (85.1%) from 2016 to 2018, respectively. The most commonly reported malaria species was *P. vivax* (490,861, 71.7%)*.* Hotspots of both *P. falciparum* and *P. vivax* were located in the border municipalities of Venezuela and Guyana. *P. falciparum* transmission was positively associated with altitude  and maximum temperature lagged at 1-month, and negatively associated with precipitation lagged at 6-months and minimum temperature without lag. Whereas *P. vivax* was positively associated with altitude and maximum temperature lagged at 2-months and negatively associated with precipitation and minimum temperature lagged at 6-months.

Since 1990, the majority of malaria cases in Venezuela have come from Bolivar state: > 60% (1992–1995) and 88% (2000–2014)^7,31–33^, with most cases clustering in Sifontes municipality (Bolivar State)^7^. Additionally, in Sifontes municipality, gold mining has been associated with a high incidence of malaria, with miners accounting for up to 80% of cases^8,9,31,34^. In contrast to progress made in neighboring countries and in the Americas, the political and economic crisis in Venezuela has thwarted malaria control efforts. Malaria cases have increased significantly in recent years: from 35,500 cases in 2000 to over 467,000 cases in 2019^2^.

Our study showed that malaria hotspots were consistently found in municipalities along the Venezuela-Guyana border with Brazil, including Sifontes municipality, and in municipalities adjacent to Sifontes municipality, including Piar, Padre Pedro Chien, Roscio, and El Callao in Bolivar state. Sifontes municipality was recently identified as the most important cluster of malaria transmission in the Americas^7^. This highlights the issue of cross-border malaria, which can impact malaria control efforts^35,36^. A plausible solution can be cross-country collaboration to improve surveillance and finding ways to provide early diagnosis and treatment for border populations, which are usually more mobile and difficult to track. Higher cases in these regions have also been related to occupation, especially gold mining^37^. Gold mining drives increased population movement to mining sites, usually young males^8^. Our findings confirmed this. Males made up two-thirds of cases and more than half of malaria cases were in the 19–40 year age group (Table 1). Furthermore, poor living conditions and working outdoors during late at night or early in the morning also could expose miners to increased mosquito bites.

*Plasmodium vivax* was the predominant species in this study and is also the primary species in the Americas^34,38^. Relapse of *P. vivax* is associated with the release of dormant hypnozoites from the liver. Challenges to correct diagnosis include lack of sensitive diagnostic tools. Rapid Diagnostic Tests (RDTs), which are widely used in the region and globally, are unable to diagnose dormant hypnozoites in the liver or in pregnant women. Secondly, adherence to *P. vivax* treatment is a main challenge, which includes a three-day course of chloroquine and 7 or 14 days of primaquine^39,40^. Hence, continued *P. vivax* transmission in other parts of world has been attributed to lack of adherence to treatment^41,42^. Since cross-border populations are hard to follow up, we propose implementation of community-based adherence support, which has been used for HIV and TB and has significantly improved treatment^43–45^. Treatment follow up can be done through a friend or family member who is travelling with the patient or someone who is part of the patient’s community such as a community member, a support group, and/or a religious leader.

Altitude was positively associated with both both species of malaria. This is partly explained by the fact that the study area is generally low-lying with altitude ranging from 64.2 to 995.5 meters. Malaria incidence decreased with increased altitude due to decreases in temperature, which makes the environment unsuitable for *Anopheles* vectors ^46–48^. Further, temperature variation influences the incidence and transmission of infection due to its direct effect on development and survivorship of vectors and malaria parasites^49^.

Climatic variables of precipitation and temperature are also associated with the transmission of malaria in this study. The transmission of the malaria parasite and mosquito survival are affected by temperature^50,51^. At temperatures of 22 °C, it takes less than three weeks to complete the life cycle of malaria parasite development in the mosquito vector^52^. The biting rate and gonotrophic processes are also temperature dependent^53,54^. Other studies have reported rainfall as an important driver of malaria transmission^55,56^. The main vectors responsible for malaria transmission in the Americas, including *Anopheles darlingi* and *An. Albimanus,* are also affected by climate^57–60^.

There are some limitations to this study. First, the main limitation is the lack of completeness and representativeness of surveillance data. Second, the true number of malaria cases could have been underestimated if cases were diagnosed and treated in private health settings or self-diagnosed and not captured by the national surveillance system. Third, populations of municipalities were projected, which may have resulted in over or under estimation. Fourth, unmeasured risk modifiers including socio-economic development, living standards, occupation, treatment, localized behavioral patterns, population mobility, and bed net use and residual indoor insecticide coverage were unaccounted for in this study. Fifth, since entomological data were not available, they were not included in the model. Entomological data would have improved the model. Hence, we suggest including these data in further analysis if available. 

## Conclusion

*Plasmodium falciparum* and *P. vivax* transmission was negatively associated with increased precipitation and minimum temperature, and positively associated with altitude and maximum temperature. Hotspots of both *P. falciparum* and *P. vivax* were isolated in eight municipalities along the Venezuela and Guyana international border with Brazil. Targeted distribution of resources, including prompt diagnosis and treatment and intensified interventions in hotspot municipalities, will be required for effective control of local malaria transmission. Furthermore, cross-border surveillance needs to be strengthened and ongoing identification of hotspots is needed to stay on track with malaria elimination targets.

## Supplementary Information

Supplementary Information.

## Data Availability

The study dataset can be made available only upon the approval by researchers and organizations involved.

## Abbreviations

AIC: Akaike’s information criterion
CAR: Conditional autoregressive
CrI: Credible interval
DIC: Deviation information criterion
RR: Relative risk
SIVEP: MalariaBrazilian Malaria Epidemiology Surveillance Information System
SMRs: Standardized morbidity ratios
VIF: Variance inflation factors
WHO: World Health Organization
