# Supplementary material for: Spatial patterns and climate drivers of malaria in three border areas of Brazil, Venezuela and Guyana, 2016–2018
Source: Sci Rep. 2022 Jun 29;12:10995. doi: 10.1038/s41598-022-14012-4 (PMC9243034; doi:10.1038/s41598-022-14012-4)
Supplement: Supplementary file 1 — Supplementary Information. [file 41598_2022_14012_MOESM1_ESM.pdf]

Spatial patterns and climate drivers of malaria in three border areas of Brazil, Venezuela and Guyana, 2016-2018

Kinley Wangdi[1*¥,] Erica Wetzler[2¥], Horace Cox[3], Paola Marchesini[4], Leopoldo Villegas[5,6], Sara Canavati[2]

[1]Department of Global Health, National Centre for Epidemiology and Population Health, ANU College of Health and Medicine, The Australian National University, Canberra, ACT 2601, Australia

[2]World Vision US, 34834 Weyerhaeuser Way South, Federal Way, Washington, USA

[3]Vector Borne Diseases Unit, Guyana Ministry of Health, Georgetown, Guyana

[4]Department of Surveillance for Zoonotic and Vector Borne Diseases, Malaria Technical Group, Ministry of Health, Brasilia, Federal District, Brazil

[5]Global Development One, Silver Spring, MD, USA

[6]Asociación Civil Impacto Social (ASOCIS), Tumeremo, Bolívar, Venezuela

*Corresponding author

[¥]Contributed equally

Email:

KW: kinley.wangdi@anu.edu.au

EW: ewetzler@worldvision.org

HC: horacecox17@yahoo.com

PM: paola.b.marchesini@gmail.com

LV: leopoldovillegas2@gmail.com

SC: saracanavati@yahoo.com

Table S1 Climatic variable selection for *Plasmodium falciparum*

Variables	IRR	p value	AIC	BIC
**Precipitation**				
No lag	0.998	<0.0001	674515.8	674525.8
One-month lag	0.998	<0.0001	671545.300	671555.300
Two months lag	0.998	<0.0001	668456.400	668466.300
Three-months lag	0.997	<0.0001	665186.600	665196.600
Four-month lag	0.997	<0.0001	662514.8	662524.7
Five-months lag	0.997	<0.0001	659744.1	659754.1
Six-months lag	0.997	<0.0001	**659353.8***	659363.7
Seven-month lag	0.997	<0.0001	662459.3	662469.3
**Minimum temperature**				
No lag	1.295899	<0.0001	**660840.000***	660850.000
One-month lag	1.290	<0.0001	661860.900	661870.900
Two months lag	1.260	<0.0001	664491.100	664501.100
Three-months lag	1.200	<0.0001	670260.600	670270.500
Four-month lag	1.17	<0.0001	672939.7	672949.7
Five-months lag	1.13	<0.0001	675413	675423
Six-months lag	1.11	<0.0001	676688.1	676698
Seven-month lag	1.07	<0.0001	678341	678350.9
**Maximum temperature**				
No lag	1.54	<0.0001	623912.600	623922.600
One-month lag	1.555015	<0.0001	**622373.400***	622383.400
Two months lag	1.540	<0.0001	624538.300	624548.300
Three-months lag	1.480	<0.0001	634759.200	634769.200
Four-month lag	1.45	<0.0001	640588.2	640598.1
Five-months lag	1.38	<0.0001	648039.2	648049.2
Six-months lag	1.37	<0.0001	649486.3	649496.2
Seven-month lag	1.3	<0.0001	658377.5	658387.4

*selected

Table S2 Climatic variable selection for *Plasmodium vivax*

Variables	IRR	p value	AIC	BIC
**Precipitation**				
No lag	0.99998	0.124	1467283	1467293
One-month lag	0.99997	<0.0001	1466769	1466779
Two months lag	0.9997	<0.0001	1465688	1465697
Three-months lag	0.9993	<0.0001	1462814	1462824
Four-month lag	0.9988	<0.0001	1457381	1457391
Five-months lag	0.999	<0.0001	1453769	1453779
Six-months lag	0.998	<0.0001	**1449901***	1449910
Seven-month lag	0.999	<0.0001	1454306	1454316
**Minimum temperature**				
No lag	0.78	<0.0001	1394461	1394471
One-month lag	0.77	<0.0001	1390713	1390723
Two months lag	0.78	<0.0001	1393519	1393529
Three-months lag	0.78	<0.0001	1394141	1394151
Four-month lag	0.76	<0.0001	1392708	1392718
Five-months lag	0.77	<0.0001	1389959	1389969
Six-months lag	0.77	<0.0001	**1388966***	1388976
Seven-month lag	0.78	<0.0001	1393242	1393252
**Maximum temperature**				
No lag	0.89	<0.0001	1454771	1454781
One-month lag	0.87	<0.0001	1448302	1448312
Two months lag	0.87	<0.0001	**1447754***	1447764
Three-months lag	0.88	<0.0001	1450183	1450193
Four-month lag	0.89	<0.0001	1452784	1452794
Five-months lag	0.7	<0.0001	1453275	1453285
Six-months lag	0.9	<0.0001	1453505	1453515
Seven-month lag	0.89	<0.0001	1451233	1451243

*Selected variable

Table S3 Collinearity assessment of selected variables for *P. falciparum*

Variables	VIF*	1/VIF
Altitude	3.99	0.250930
Precipitation lagged six months	1.17	0.853569
Minimum Temperature no lag months	4.71	0.212092
Maximum Temperature lagged one month	2.19	0.457527
Mean VIF	3.01	

*VIF- variance inflation factors

Table S4 Collinearity assessment of selected variables for *P. vivax*

Variables	VIF*	1/VIF
Altitude	2.32	0.430929
Precipitation lagged six months	1.19	0.50955
Minimum Temperature lagged six months	2.12	0.471227
Maximum Temperature lagged two months	1.96	0.509955
Mean VIF	1.90	

*VIF- variance inflation factors

Table S5 Summary table of climatic variables

Climatic variable	Mean	Standard deviation	Minimum	Maximum
Altitude	283.5	198.0	64.2	995.5
Precipitation	150.5	107.6	1.1	413.6
Minimum temperature	21.9	1.4	16. 9	24.6
Maximum temperature	31.1	1.3	26.8	35.2
